# Functional Validation of *CLDN* Variants Identified in a Neural Tube Defect Cohort Demonstrates Their Contribution to Neural Tube Defects

**DOI:** 10.3389/fnins.2020.00664

**Published:** 2020-07-14

**Authors:** Amanda I. Baumholtz, Patrizia De Marco, Valeria Capra, Aimee K. Ryan

**Affiliations:** ^1^Department of Human Genetics, McGill University, Montreal, QC, Canada; ^2^The Research Institute of the McGill University Health Centre, Montreal, QC, Canada; ^3^Laboratorio di Neurogenetica e Neuroscienze, Istituto Giannina Gaslini, Genoa, Italy; ^4^U.O. Neurochirurgia, Istituto Giannina Gaslini, Genoa, Italy; ^5^Department of Pediatrics, McGill University, Montreal, QC, Canada

**Keywords:** claudin, tight junction, neural tube defects, myelomeningocele, fluidigm

## Abstract

Neural tube defects (NTDs) are severe malformations of the central nervous system that affect 1–2 individuals per 2,000 births. Their etiology is complex and involves both genetic and environmental factors. Our recent discovery that simultaneous removal of Cldn3, -4, and -8 from tight junctions results in cranial and spinal NTDs in both chick and mouse embryos suggests that claudins play a conserved role in neural tube closure in vertebrates. To determine if claudins were associated with NTDs in humans, we used a Fluidigm next generation sequencing approach to identify genetic variants in *CLDN* loci in 152 patients with spinal NTDs. We identified eleven rare and four novel missense mutations in ten *CLDN* genes. *In vivo* validation of variant pathogenicity using a chick embryo model system revealed that overexpression of four variants caused a significant increase in NTDs: *CLDN3* A128T, *CLDN8* P216L, *CLDN19* I22T, and E209G. Our data implicate rare missense variants in *CLDN* genes as risk factors for spinal NTDs and suggest a new family of proteins involved in the pathogenesis of these malformations.

## Introduction

Neural tube defects (NTDs) [MIM: 182940] are a group of congenital central nervous system malformations that affect the brain and spinal cord, caused by failure of neural tube closure during embryogenesis. They have a complex etiology involving both genetic and environmental factors. The two most common forms are the cranial defect, anencephaly, and the spinal defect, myelomeningocele (spina bifida). A third rare form of open NTDs, craniorachischisis, occurs when the neural tube fails to close along the entire length of the body axis. NTDs are the second most common birth defect with a varying incidence, affecting 1–2 per 2,000 pregnancies in North America post-folic acid fortification, and as high as 20 per 1,000 births in certain regions of China (Chen et al., 2009). Approximately 300,000 newborns affected by these defects are born annually (Zaganjor et al., 2016). Thus, there is an urgent need to better understand the underlying pathogenic mechanisms of NTDs, particularly those not prevented by folic acid fortification.

Neural tube closure, which occurs during the fourth week of human gestation, is divided into four distinct phases (Wang et al., 2019). First, a subset of medial ectodermal cells is induced to differentiate into neural progenitors, which form the neural plate. Next, the neural plate undergoes convergent extension, which leads to its mediolateral narrowing and anterior–posterior extension. This is followed by apical constriction of midline cells to form the median hinge point, which allows the neural plate to bend at the midline and the lateral edges of the neural plate to elevate and become the neural folds. Finally, the lateral edges of the neural folds meet at the dorsal midline where they fuse to generate a closed neural tube and a contiguous overlying layer of surface ectoderm.

A defect in any of these phases of neural tube closure will result in a NTD. Defects in convergent extension, which is regulated by the non-canonical Wnt/planar cell polarity (PCP) pathway, result in NTDs due to the fact that the lateral edges of the neural folds never come in close enough proximity to fuse. Many animal models with mutations in PCP components develop NTDs due to aberrant convergent extension movements (Greene et al., 1998; Goto and Keller, 2002; Wallingford and Harland, 2002; Curtin et al., 2003; Wang J. et al., 2006; Wang Y. et al., 2006; Ybot-Gonzalez et al., 2007; Paudyal et al., 2010) and rare and novel missense mutations in PCP genes are risk factors for NTDs in humans (Kibar et al., 2009, 2011; Lei et al., 2010, 2019; Bosoi et al., 2011; Allache et al., 2012; De Marco et al., 2012, 2013; Robinson et al., 2012). The PCP pathway is one of the few candidate pathways identified in animal models whose human homologs have been shown to contribute to risk of human NTDs. Formation of the hinge points requires RhoA/ROCK-mediated actin-myosin contraction of the apical surface of neural plate cells (Kinoshita et al., 2008; Rolo et al., 2009; Escuin et al., 2015).

We recently discovered that claudins act upstream of the PCP and RhoA/ROCK signaling pathways to regulate cell shape changes and tissue movements during neural tube morphogenesis (Baumholtz et al., 2017). Indeed, simultaneous removal of Cldn3, -4, and -8 from tight junctions causes open NTDs in both chick and mouse embryos (Baumholtz et al., 2017). Claudins comprise a family of integral tight junction proteins with ∼24 unique members in vertebrates. They have four transmembrane domains, two extracellular loops, and cytoplasmic N- and C-termini. The first extracellular loop contains charged amino acids that determine the ion and size selectivity of the tight junction paracellular barrier (Katahira et al., 1997; Hamazaki et al., 2002; Simard et al., 2005; Robertson et al., 2007; Baumholtz et al., 2017; Nakamura et al., 2019). The second extracellular loop participates in *trans*-interactions with claudin molecules in apposing cells, and in *cis*-interactions with other claudins in the same cell (Piontek et al., 2008; Suzuki et al., 2014). The transmembrane domains also participate in *cis*-oligomerization (Rossa et al., 2014). Together, these interactions constitute the backbone of tight junction strands and prevent the mixing of apical and basolateral proteins (Krause et al., 2015). The claudin cytoplasmic C-terminal tail has a conserved PDZ-binding domain that interacts with adaptor proteins at the tight junction cytoplasmic plaque, thereby linking the tight junction to the actin cytoskeleton and intracellular signaling events (Van Itallie et al., 2013; Fredriksson et al., 2015; Baumholtz et al., 2017). The C-terminal domain also contains numerous phosphorylation sites that can influence protein interactions at the tight junction cytoplasmic plaque (Tanaka et al., 2005; Ikari et al., 2006).

Given the importance of claudins in neural tube closure in chick and mouse, we tested our hypothesis that mutations in *CLDN* genes are risk factors for human NTDs by screening a cohort of 152 open spinal NTD patients for rare and novel non-synonymous *CLDN* variants. We identified 11 rare and four novel missense variants in 10 *CLDN* genes. We then tested the functional consequences of the *CLDN* variants by transfection of HEK293 or MDCK II cells to determine if the variant protein localized to the tight junction and then by overexpressing the variants in chick embryos to determine if the variants caused defects in neural tube closure. Our data suggest that rare missense mutations in *CLDN* genes are risk factors for human NTDs.

## Subjects and Methods

### Neural Tube Defect Cohort

The studies involving human participants were reviewed and approved by IRCCS Istituto Giannina Gaslini (Protocol number: IGG-VACA, 18 September 2011) and the Research Institute of the McGill University Health Centre (Protocol number: 14-444-PED). Written informed consent to participate in this study was provided by the participants and/or their legal guardian/next of kin. The patient cohort consisted of 152 unrelated individuals with myelomeningocele, who were recruited at the Spina Bifida Center of the Gaslini Hospital in Genova, Italy, between the period of 2006–2017. The age of patients ranged from 3 to 20 years, with the mean age being 9.6 years. The female/male ratio was 1.2. All participants were Italians with antecedents from all parts of the country. The majority of these individuals were of European ancestry, although some individuals were of Hispanic or African ancestry. Upon entering the study, individuals were re-evaluated by a clinical geneticist and diagnosis was made on the basis of MRI, X-ray images and clinical records, according to previously described criteria (Rossi et al., 2004). The study group includes individuals who were previously analyzed for mutation of PCP genes (Kibar et al., 2009, 2011; Bosoi et al., 2011; Allache et al., 2012; De Marco et al., 2012, 2013; Robinson et al., 2012).

### Next-Generation DNA Sequencing

Genomic DNA was isolated from blood samples using the QIAamp DNA blood kit according to the manufacturer’s protocol (Qiagen, Milan, Italy). The genomic sequence of *CLDN*s was determined using the UCSC Genome Browser assembly ID hg19 (*CLDN1*: RefSeq NG_021418.1 [MIM: 607626]; *CLDN2*: RefSeq NG_016445.1, *CLDN3*: RefSeq NG_012023.1; *CLDN4*: RefSeq NG_012868.1; *CLDN5*: GenBank NC_000022.10; *CLDN6*: GenBank *N*C_000016.9; *CLDN7*: GenBank *N*C_000017.10; *CLDN8*: RefSeq NG_050758.1; *CLDN9*: NC_000016.9; *CLDN10*: RefSeq NG_047100.1 [MIM: 617671]; *CLDN11*: GenBank NC_000003.11; *CLDN12*: GenBank NC_000007.13; *CLDN14*: GenBank NC_000021.8 [MIM: 614035]; *CLDN15*: GenBank NC_000007.13; *CLDN16*: RefSeq NG_008149.1 [MIM: 248250]; *CLDN17*: GenBank NC_000021.8; *CLDN18*: GenBank NC_000003.11; *CLDN19*: RefSeq NG_008993.1 [MIM: 248190]; *CLDN20*: RefSeq NG_027528.2; *CLDN21*: RefSeq NG_051586.1; *CLDN22*: RefSeq NG_051586; *CLDN23*: GenBank NC_000008.10; *CLDN24*: RefSeq NG_051586.1; *CLDN25*: GenBank NC_000011.9). DNA was then processed by microfluidic polymerase chain reaction (PCR) using a custom Fluidigm Access Array, followed by next-generation sequencing on an Illumina MiSeq platform at the McGill University and Genome Quebec Innovation Center (Montreal, QC, Canada). Briefly, 92 custom pairs of primers were designed to amplify ∼300 bp fragments of the coding exons of the 24 human claudin genes (Supplementary Table S1). Primer sets were validated using a standard PCR protocol to ensure that they amplified a single fragment of the expected size, and selected amplicons were validated by Sanger sequencing. The amplicons from the array were pooled and the resulting pools were multiplexed and sequenced using the Illumina MiSeq. Reads were processed and aligned to the human genome (UCSC hg19, NCBI build 37) by Genome Quebec. Aligned and processed BAM files were used to manually identify variants using the Integrative Genomics Viewer version 2.3 (Robinson et al., 2011, 2017; Thorvaldsdottir et al., 2013). Variants with alternative allele frequencies of 30% or greater were selected for further analysis.

### Sanger Sequencing of CLDN3

The single *CLDN3* coding exon was amplified by PCR using a single primer pair (Supplementary Table S2) and subjected to Sanger sequencing at the McGill University and Genome Quebec Innovation Centre (Montreal, QC, Canada). Samples were sequenced in both directions using specific forward and reverse primers. The *CLDN3* variants were confirmed by repeating the PCR and re-sequencing from a single direction.

### Bioinformatics

Variants were annotated according to the HGVS nomenclature^1^. The Exome Variant Server (EVS^2^ (Exome Variant Server, 2020) and Genome Aggregation Database v2.1.1 (gnomAD^3^ (Karczewski et al., 2020) public databases were queried for the presence and incidence of the variants identified in *CLDN1-25*. Rare variants were defined as having a minor allele frequency (MAF) < 1% in these databases and novel variants were defined as those not present in these databases. The potential pathogenic effect of the identified mutations on protein function was predicted using three software programs: Polymorphism Phenotyping v2 (PolyPhen-2^4^; Adzhubei et al., 2010), Sorting Intolerant from Tolerant (SIFT^5^) (Ng and Henikoff, 2003) and MutationTaster^6^ (Schwarz et al., 2010). Multiple alignments of the CLDN proteins were done using the Clustal Omega program^7^. Localization of the variants in protein domains was assessed by Uniprot^8^. Predicted phosphorylation sites in CLDN proteins were identified using NetPhos 3.1^9^ (Blom et al., 1999).

### DNA Resequencing of *CLDNs*

Rare and novel missense variants were validated by Sanger sequencing. Primers flanking the coding exons of *CLDN3* (RefSeq NM_001306.3), *CLDN4* (RefSeq NM_001305.4), *CLDN6* (RefSeq NM_021195.4), *CLDN8* (RefSeq NM_199328.2), *CLDN9* (RefSeq NM_020982.3), *CLDN14* (RefSeq NM_144492.2), *CLDN16* (RefSeq NM_006580.3), *CLDN18* (RefSeq NM_001002026.2), *CLDN19* (RefSeq NM_148960.2), *CLDN23* (RefSeq NM_194284.2), *CLDN24* (RefSeq NM_001185149.1) were used to amplify the coding region by polymerase chain reaction (Supplementary Table S2). Amplicons were subjected to Sanger sequencing at the McGill University and Genome Quebec Innovation Centre (Montreal, QC, Canada).

### Generation of Claudin Wild-Type and Mutant Constructs

The full-length human wild-type and variant sequences for claudins encoded by a single exon (*CLDN3*, *4*, *6*, and *24*) were amplified from genomic DNA of NTD patients by PCR and cloned into the pSCA vector using the Stratagene PCR cloning kit (Agilent Technologies, Santa Clara, CA, United States). pMXs-Puro-CLDN18 was a gift from Axel Hillmer (Addgene plasmid #69466) (Yao et al., 2015) and used as a template to clone full-length CLDN18 into pSCA. *CLDN16* p.N223S, *CLDN18* p.V88I and *CLDN19* p.I22T were introduced in the pSCA-claudin constructs by site-directed mutagenesis using the Stratagene QuikChange II Site-Directed Mutagenesis kit according to manufacturer’s directions (Agilent Technologies, Santa Clara, CA, United States). *CLDN19* p.E209G was created by PCR using a reverse primer containing the mutation and pSCA-HCLDN19 as a template. Sequence identity of cDNA clones was confirmed by sequencing at the McGill University and Genome Quebec Innovation Centre (Montreal, QC, Canada). Wild-type and variant constructs were then cloned into the pCanHA3 and pMES-IRES-GFP expression plasmids. Primer sequences used for mutagenesis reactions are shown in Supplementary Table S3.

### Immunolocalization of Ectopically Expressed Claudins

HEK293 and MDCK II cells were grown in Dulbecco’s Modified Eagle’s Medium supplemented with 10% fetal bovine serum and antibiotics. HEK293 cells were plated on coverslips in a 24-well plate and reached 70–90% confluence the day of the transfection experiments. pMES or pCanHA3 expression vectors encoding wild-type or variant claudins were transiently transfected into HEK293 cells using Lipofectamine LTX (Invitrogen) in a 1:1 DNA-reagent ratio using 500 ng of plasmid DNA per well. MDCK II cells were transfected in suspension using Polyjet (FroggaBio) in a 4:1 DNA-reagent ratio using 500 ng plasmid per well and plated on coverslips in a 24-well plate at 70% confluency. HEK293 and MDCK II cells were cultured for 24 h after transfection. After rinsing with PBS, cells were fixed with 10% trichloroacetic acid or 4% PFA for 10 min at 4°C, blocked with 10% normal goat serum in 0.3% Triton X-100 in PBS and then incubated with primary antibody for 1 h at room temperature. Primary antibodies used were Cldn3 and 16 (Abcam, Cambridge, United Kingdom, 1:100), Cldn8 (Invitrogen, Carlsbad, CA, United States, 1:100), ZO-1 (Invitrogen, 1:100, Carlsbad, CA, United States), and HA (Invitrogen, Carlsbad, CA, United States, 1:100). Antibodies were detected with Alexa Fluor^®^ 488 conjugated goat anti-rabbit IgG and Alexa Fluor^®^ 594 conjugated goat anti-mouse IgG (Molecular Probes). Slides were coverslipped with SlowFade Gold Antifade kit (Molecular Probes), which contained DAPI to enable visualization of the nuclei, and imaged using a laser scanning confocal microscope (LSM780, Leica, Germany).

### Overexpression Assay in the Chick Embryo

CLDN-IRES-GFP pMES expression vectors (5 μg/μl) were injected into the space between the vitelline membrane and the epiblast of HH4 embryos cultured *ex ovo* on agar-albumen plates using a Narishige IM 300 microinjector, and then electroporated using a Protech CUY21SC square electroporator (five 3 V/50 ms pulses at 500 ms intervals). Manipulated embryos were cultured at 39.5°C until HH11-13 and then scored for defects in neural tube closure and convergent extension. Brightfield and GFP images of electroporated embryos were taken using a Leica M205FA dissecting microscope and Leica DFC450C camera with Leica Application Suite X software. Only embryos with GFP expression in neural and non-neural ectoderm cells were included for phenotype evaluation.

### Assessment of Axial Length

Dorsal images of HH12 embryos (somite stage 15–17) were photographed using a Leica M125 dissecting microscope and imported into ImageJ software. Axial length was measured from the tip of the forebrain to the tip of the tail bud.

### Whole Mount *in situ* Hybridization

Whole-mount *in situ* hybridization was performed as previously described (Collins and Ryan, 2011). Embryos were photographed using a Leica M125 dissecting microscope with Infinity Capture software v5.0.2 (Lumenera Corp.). Paraffin-embedded embryos were sectioned and photographed using a Zeiss Axiophot compound microscope and AxioCamMRc camera with Axiovision v4.7.1.0 software.

### Histology

Embryos were fixed overnight in 4% paraformaldehyde, dehydrated in a graded ethanol series and embedded in paraffin wax. Seven-micron sections were stained using Mayer’s hematoxylin solution and Eosin Y (both Sigma). Images were captured on a Leica DM6000B upright microscope equipped with a DFC450C camera.

### Statistical Analyses

Anterior–posterior length of embryos overexpressing variant *CLDN* constructs compared to wild-type *CLDN* was analyzed by Mann–Whitney *U*-test. To assess the ability of *CLDN* variants to affect neural tube closure in our gain-of-function experiments, a Chi-square test was performed comparing the proportion of open NTDs between embryos overexpressing *CLDN* variants compared to wild-type *CLDN* or control empty plasmid. All statistics were computed using GraphPad Prism software.

## Results

### NTD Patients Have Rare and Novel *CLDN* Variants

To examine the association of *CLDN* mutations in the etiology of human NTDs, we sequenced the coding region of the 24 human *CLDN* genes in 152 patients with open spinal NTDs (myelomeningocele) by targeted next generation sequencing. Recent publications of large population sequencing data provide an opportunity to characterize with accuracy and precision the frequency distributions of rare disease-causing alleles. Since we did not have an adequate control group for size and for ethnicity, we decided to use the Exome Variant Server (EVS) (Exome Variant Server, 2020) that catalogs whole exome sequencing data of 6503 unrelated European and African Americans, and the gnomAD database (Karczewski et al., 2020) that provides variants from 125,748 exomes and 15,708 genomes of unrelated individuals for making inferences on the overall role of *CLDN* variants. Importantly, neither database contains individuals with severe congenital anomalies. The EVS database contains healthy controls and extremes of specific adult onset traits and/or diseases, including blood pressure, early onset myocardial infarction and stroke, and lung disease. In the gnomAD database, individuals affected by severe pediatric disease, and their first-degree relatives, were removed. Thus, neither database contains individuals with myelomeningocele open NTDs, supporting their use as appropriate control populations for our study cohort. The individuals in these databases are adults, and therefore, deleterious variants for any gene that have the potential to contribute to conditions associated with early mortality may be underrepresented. To date, gnomAD is the largest and most comprehensive source of genetic information of individuals of different ethnic backgrounds and similar to our cohort, the largest ethnicity represented is non-Finnish Europeans. However, neither database provides precise ancestry information. Another potential limitation to the database is that the sequencing data are derived from different exome capture approaches and sequencing platforms, which leads to variation in coverage between individuals and across sites. While this may impact the exact allele frequency, the large number of individuals that have been analyzed allows us to distinguish between common, rare and novel variants. With respect to our own data, we only considered variants where the alternate allele was present in at least 30% of the sequencing reads.

**FIGURE 1:**
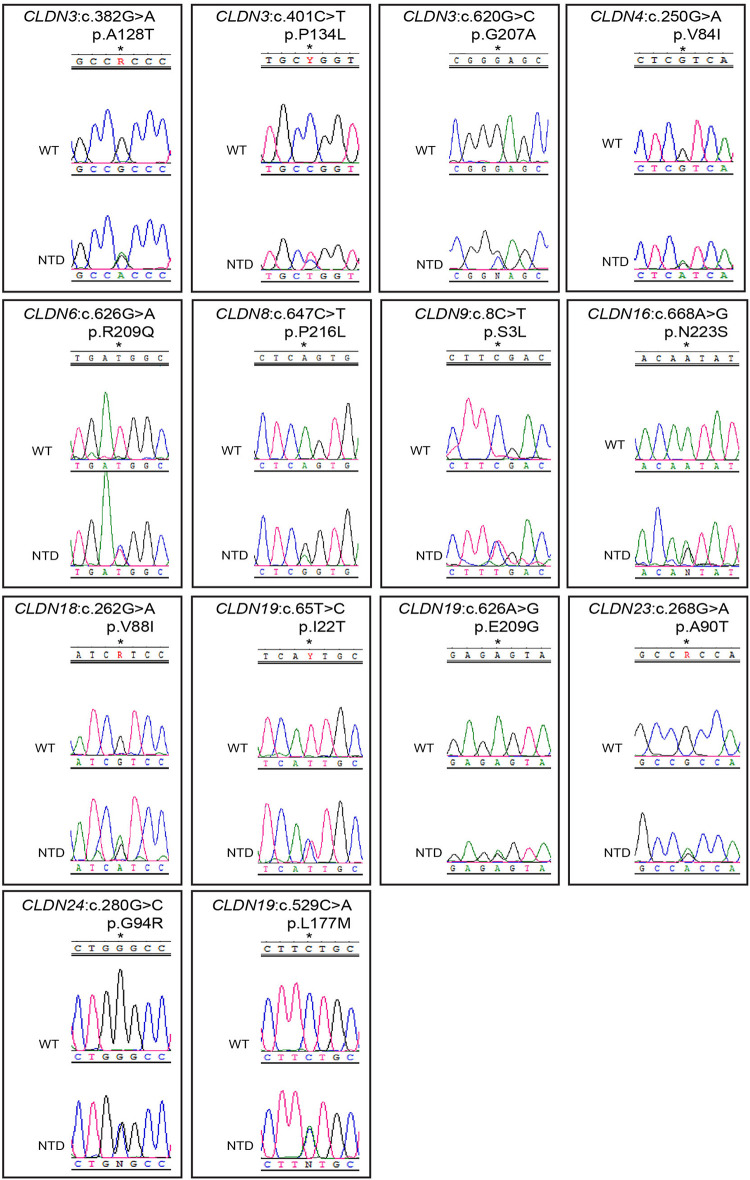
Chromatograms of rare and novel non-synonymous *CLDN* mutations in NTD patients and absent in controls. Mutations are shown in the sense (5′ → 3′) direction. The position of altered amino acids are indicated with an asterisk. WT, wild-type sequence; NTD, patient sequence.

We found 69 variants in total: 37 synonymous single nucleotide polymorphisms (SNPs) and 32 non-synonymous missense variants. Of the missense variants, 17 were common (Table 1), 11 were rare [minor allele frequency (MAF) < 1%] and four were not found in the dbSNP (Sherry et al., 2001), EVS (Exome Variant Server, 2020), or gnomAD (Karczewski et al., 2020) publicly available databases (Table 2). All rare and novel missense variants in *CLDN*s were confirmed by Sanger sequencing, except for the *CLDN24* p.E161K as there was no genomic DNA remaining for this patient (Figure 1). All rare and novel missense variants discovered were heterozygous and most were found in a single patient with the exception of *CLDN3* p.A128T that was found in three unrelated patients and *CLDN3* p.P134L, *CLDN6* p.R209Q and *CLDN18* p.V88I that were each found in two unrelated patients (Table 2). No NTD patient was a carrier of a rare or novel missense mutation in more than one *CLDN* gene. Sixteen of the 20 patients with a novel or rare mutation in a *CLDN* gene were previously analyzed for mutations in PCP genes (Kibar et al., 2009, 2011; Bosoi et al., 2011; Allache et al., 2012; De Marco et al., 2012, 2013; Robinson et al., 2012). A mutation in a PCP gene was identified in 4 of these 16 patients (Table 3); mutations in PCP genes have been associated with NTDs.

**TABLE 1:** Common non-synonymous variants in the coding sequence of *CLDN1-25*.

Gene	Accession	Nucleotide	Amino acid	Allele frequency count/	Allele frequency count/	Allele frequency
	number	change	change	(%) NTD cases	(%) gnomAD	count/(%) EVS
*CLDN6*	NC_000016.9	c.388G > C	p.V130L	1 (0.33)	3938 (1.396)	161 (1.2388)
		c.427A > G	p.I143V	126 (41.45)	101902 (36.36)	5010 (38.5741)
*CLDN7*	NC_000017.10	c.590T > C	p.V197A	212 (69.74)	185318 (65.8)	3944 (30.3245)
*CLDN8*	NG_050758.1	c.290T > C	p.M97T	1 (0.33)	1608 (0.5687)	229 (1.7607)
		c.385A > G	p.T129A	20 (6.57)	14671 (5.192)	1103 (8.4807)
		c.451T > C	p.S151P	76 (25)	84042 (29.83)	8008 (38.4284)
*CLDN14*	NC_000021.8	c.11C > T	p.T4M	14 (4.60)	4972 (2.738)	402 (4.3946)
*CLDN16*	NG_008149.1	c.C166del	p.A56LfsTer16	116 (38.16)	54308 (19.24)	0 (0)
		c.166C > G	p.A56P	116 (38.16)	54311 (19.24)	0 (0)
*CLDN17*	NC_000021.8	c.244G > A	p.A82T	31 (10.20)	26892 (9.437)	892 (6.8584)
*CLDN18*	NC_000003.11	c.445A > T	p.M149L	19 (6.25)	32284 (11.42)	1398 (10.7489)
*CLDN19*	NG_008993.1	c.599G > A	p.R200Q	2 (0.66)	3396 (1.508)	148 (1.1422)
*CLDN20*	NG_027528.2	c.281G > A	p.G94E	2 (0.66)	3977 (1.406)	119 (0.915)
*CLDN23*	NC_000008.10	c.628G > A	p.V210M	28 (9.21)	26931 (13.32)	941 (7.8521)
*CLDN24*	NG_051586.1	c.52C > T	p.L18F	222 (73.03)	208872 (76.22)	N/A
		c.391A > G	p.I131V	2 (0.66)	3227 (1.163)	N/A
		c.621G > C	p.Q207H	55 (18.09)	43187 (23.47)	N/A

**TABLE 2:** Novel and rare non-synonymous variants in the coding sequence of *CLDN1-25.*

Gene	Accession number	Nucleotide change	Amino acid change	Fluidigm allele frequency	Fluidigm read depth	Allele frequency count/(%) NTD cases	Allele frequency count/(%) gnomAD	Allele frequency count/(%) EVS	Ethnicity
*CLDN3*	NG_012023.1	c.382G > A	p.A128T	N/A	N/A	3 (1.0)	0 (0)	0 (0)	European
		c.401C > T	p.P134L	N/A	N/A	2 (0.7)	1253 (0.4519)	63 (0.484)	European
		c.620G > C	p.G207A	N/A	N/A	1 (0.3)	0 (0)	0 (0)	European
*CLDN4*	NG_012868.1	c.250G > A	p.V84I	49%	48282	1 (0.3)	6 (0.002388)	1 (0.0077)	European
*CLDN6*	NC_000016.9	c.626G > A	p.R209Q	54%; 53%	543; 4993	2 (0.7)	299 (0.1406)	20 (0.1539)	European, Hispanic
*CLDN8*	NG_050758.1	c.647C > T	p.P216L	55%	1293	1 (0.3)	906 (0.3204)	151 (1.161)	African
*CLDN9*	NC_000016.9	c.8C > T	p.S3L	48%	844	1 (0.3)	311 (0.1114)	3 (0.0231)	European
*CLDN16*	NG_008149.1	c.668A > G	p.N223S	40%	50	1 (0.3)	90 (0.03181)	3 (0.0231)	European
*CLDN18*	NC_000003.11	c.262G > A	p.V88I	51%; 50%	35; 1077	2 (0.7)	568 (0.2009)	22 (0.1692)	European
*CLDN19*	NG_008993.1	c.65T > C	p.I22T	50%	3067	1 (0.3)	117 (0.05170)	8 (0.0616)	European
		c.626A > G	p.E209G	43%	2407	1 (0.3)	17 (0.007741)	0 (0)	European
*CLDN23*	NC_000008.10	c.268G > A	p.A90T	57%	54	1 (0.3)	1225 (0.4753)	59 (0.4624)	European
*CLDN24*	NG_051586.1	c.280G > C	p.G94R	52%	787	1 (0.3)	0 (0)	N/A	European
		c.481G > A	p.E161K	32%	964	1 (0.3)	1 (0.0004875)	N/A	European
		c.529C > A	p.L177M	40%	1762	1 (0.3)	0 (0)	N/A	European

**TABLE 3:** Patients carrying mutations in a *CLDN* gene and core PCP gene.

CLDN gene	CLDN variant	PCP gene	PCP variant	Ethnicity
*CLDN3*	p.A128T	*CELSR1*	p.G934R	European
*CLDN6*	p.R209Q	*VANGL1*	p.C145C	European
*CLDN16*	p.N223S	*DVL2*	p.T535I	European
*CLDN23*	p.A90T	*DVL2*	p.T535I	European

### Predictions of the Impact of Rare and Novel Variants on Protein Function

Rare variants are known to be causal genetic factors for complex diseases (Manolio et al., 2009; Bruel et al., 2017) and rare missense variants have been associated with NTDs in humans (Li et al., 2018). Therefore, we focused our analysis on the 4 novel and 11 rare missense variants. PolyPhen-2 (Adzhubei et al., 2010), SIFT (Ng and Henikoff, 2003), and MutationTaster (Schwarz et al., 2010) were used to evaluate the potential pathogenic effect of each variant based on the degree of evolutionary conservation of the affected residue and the nature of the amino acid replacement and its possible impact on protein function and localization (Supplementary Table S4). 10 of the 15 rare and novel missense variants were predicted to be pathogenic with the potential to impact protein function by at least one of these tools. These variants are described below and were the focus of our functional studies.

The MAF (%) of these variants in the gnomAD and EVS databases, respectively, were 0.4519 and 0.4845 for *CLDN3* p.P134L, 0.3204 and 1.161 for *CLDN8* p.P216L, 0.1114 and 0.0231 for *CLDN9* p.S3L, 0.03181 and 0.0231 for *CLDN16* p.N223S, 0.2009 and 0.1692 *CLDN18* p.V88I, 0.0517 and 0.0616 for *CLDN19* p.I22T, 0.007741 and not reported for *CLDN19* p.E09G, and 0.0004875 and not reported for *CLDN24* p.E161K (Table 2). With the exception of the *CLDN8* p.P216L variant, ethnicity did not impact the MAF such that it would change the categorization of a variant as common versus rare (<1%). In the gnomAD database the MAF was 10-fold higher among Africans (MAF = 3.3%), and in the EVS database the MAF was 3.43% for African Americans and 0 for European Americans. The amino acid residues *CLDN3* p.P134, *CLDN16* p.N223, *CLDN18* p.V88, *CLDN19* p.E209, and *CLDN24* p.E161 are evolutionarily conserved, while *CLDN8* p.P216, *CLDN9* p.S3, and *CLDN19* p. I22 are conserved in mammals.

**FIGURE 2:**
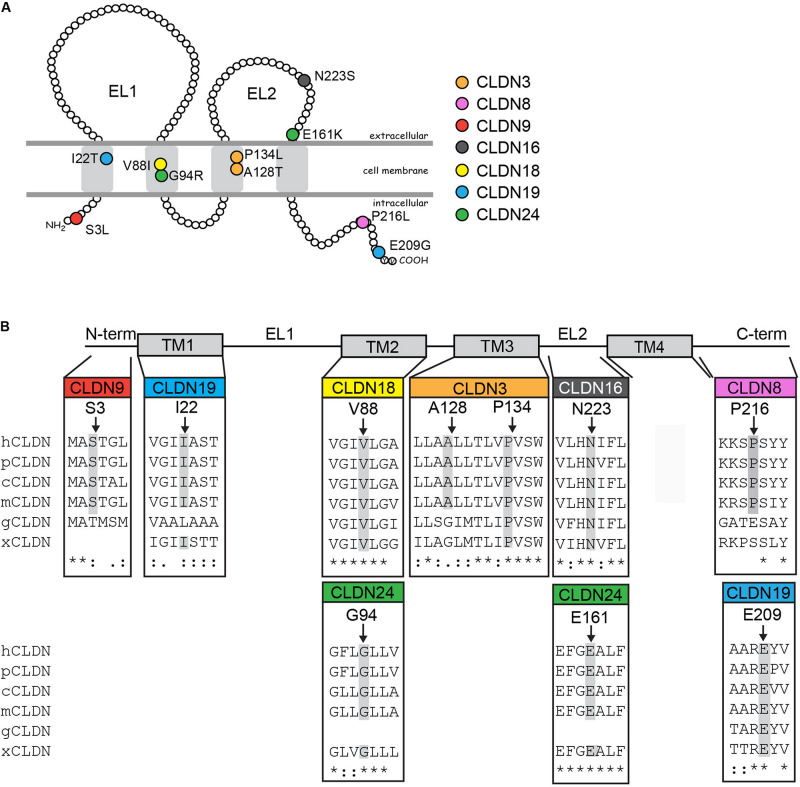
Rare and novel *CLDN* mutations in NTD patients and conservation of substituted residues. **(A)** Schematic of a claudin protein in the context of the apical lateral membrane. The approximate positions of mutations identified are shown. **(B)** Partial ClustalW protein sequence alignment of human CLDNs at the positions of the identified substitutions in the CLDN with a subset of vertebrate orthologs. Abbreviations are as follows: h, *Homo sapiens*; p, *Pan troglodytes*; c, *Canis lupus familiaris*; m, *Mus musculus*; g, *Gallus gallus*; x, *Xenopus*. The amino acid position of CLDN variants found in NTD patients are indicated by arrows and residues conserved at this position in other species are highlighted in gray. *Homo sapiens* accession numbers: *CLDN3*, NP_001297.1; CLDN8, NG_050758.1; CLDN9, NP_066192.1; *CLDN16*, NP_006571.1; *CLDN18*, AAF26448.1; *CLDN19*, Q8N6F1.2; *CLDN24*, NP_001172078.1. *Pan troglodytes*: *CLDN3*, XP_016813082.1; *CLDN8*, XP_531532.1; *CLDN9*, XP_016784715.1: *CLDN16*, XP_001161102.2; *CLDN18*, XP_526318.3; *CLDN19*, XP_003949437.2; CLDN24, XP_003310625.1. *Canis lupus familiaris*: *CLDN3*, NP_001003088.1; *CLDN8*, XP_005638865.1; *CLDN9*, XP_005621712.1: *CLDN16*, XP_850268.2; *CLDN18*, XP_534274.2; *CLDN19*, XP_848612.2; *CLDN24*, XM_540033.4. *Mus musculus*: *Cldn3*, NP_034032.1; *Cldn8*, NM_018778.3; *Cldn9*, NP_064689.2; *Cldn16*, NP_444471.1; *Cldn18*, AAF26447.1; *Cldn19*, NP_694745.1; *Cldn24*; NP_001104788.1. *Gallus gallus*: *CLDN3*, NP_989533.1; *CLDN8*, XP_004938436.1; *CLDN9*, XP_004946475.1; *CLDN16*, XP_426702.1; *CLDN18*, XP_003641778.1; *CLDN19*, XP_003642589.2. *Xenopus*: *Cldn3*, NP_001087400.1; *Cldn8*, XM_004912158.4; *Cldn16*, XP_002934087.1; *Cldn18*, NP_001083443.1; *Cldn19*, XP_002937080.1; *Cldn24*, XP_018098209.1.

These variants are located throughout the different claudin protein domains (Figure 2). *CLDN9* p.S3L localizes to the N-terminus. The amino acid substitution of serine to leucine results in loss of a putative phosphorylation site as predicted by NetPhos3.1 and could alter protein structure. The *CLDN3* p.P134L, *CLDN18* p.V88I, and *CLDN19* p.I22T variants are located in transmembrane domains. The amino acid substitution proline to leucine results in loss of a bulky side chain, which could alter the structure of the CLDN3 protein. In fact, a recent study showed that the cyclic structure of proline’s side chain at position 134 of CLDN3 causes bending of the third transmembrane domain affecting Cldn–Cldn *cis-*interactions and loss of a cyclic side chain by alanine or glycine substitution causes a change in CLDN3 protein conformation (Nakamura et al., 2019). Substituting valine with isoleucine in CLDN8 does not change the hydrophobic characteristics of the residue, although isoleucine has a bulky side chain that could interfere with protein–protein interactions. In CLDN19, replacement of isoleucine with threonine results in loss of hydrophobicity at this position and may affect the ability of the transmembrane domain to insert into the plasma membrane. *CLDN19* p.I22T also introduces a putative phosphorylation site as predicted by NetPhos3.1. *CLDN16* p.N223S and *CLDN24* p.E161K change residues in the second extracellular loop. Although an asparagine to serine substitution is considered a conservative substitution with respect to charge, NetPhos3.1 predicts that introducing a serine at *CLDN16* p.N223 creates a putative phosphorylation site. A glutamic acid to lysine switch results in a change from a positive to a negatively charged residue. Finally, two of the variants affect amino acids in the C-terminal cytoplasmic domain. The proline to leucine substitution in *CLDN8* p.P216L introduces a hydrophobic side chain, while the glutamic acid to glycine substitution in the *CLDN19* p.E209G variant results in loss of a negatively charged amino acid and is considered a non-conservative change. Given that the claudin cytoplasmic C-terminal domain interacts with adaptor proteins that bridge the tight junction to the actin cytoskeleton, these variants have the potential to disrupt protein–protein interactions that regulate morphogenetic movements and cell shape changes during neural tube closure. Both *CLDN19* variants were present in the heterozygous state in the patients’ mothers (Supplementary Figure S1). Genomic DNA was not available for the parents of the NTD patients with the *CLDN8* p.P216L, *CLDN9* p.S3L, *CLDN16* p.N223S, *CLDN18* p.V88I, and *CLDN24* p.E161K variants.

*CLDN3* p.A128T and *CLDN24* p.G94R were not present in either the gnomAD or the EVS databases. The novel p.A128T variant was found in three unrelated NTD patients. The father of one patient and the mother of another patient were heterozygous for this allele (Supplementary Figure S1). Parental DNA was not available for the third patient. The patient that inherited the *CLDN3* p.A128T variant from their father also had a rare heterozygous mutation in the PCP gene *CELSR1* p.G934R [MIM: 604532] that is predicted to be benign (Allache et al., 2012). Parental DNA was not available for genetic testing for the single NTD patient with the *CLDN24* p.G94R variant. Both of these variants localize to transmembrane domains. An alanine to threonine substitution results in reduced hydrophobicity and may affect the ability of the protein to insert into the membrane, but does not create a putative phosphorylation site in CLDN3. The substitution of a glycine to arginine in CLDN24 introduces a positively charged bulky side chain that may have structural consequences.

### *CLDN* Variants Do Not Affect the Ability of the Protein to Localize to Tight Junction Strands

**FIGURE 3:**
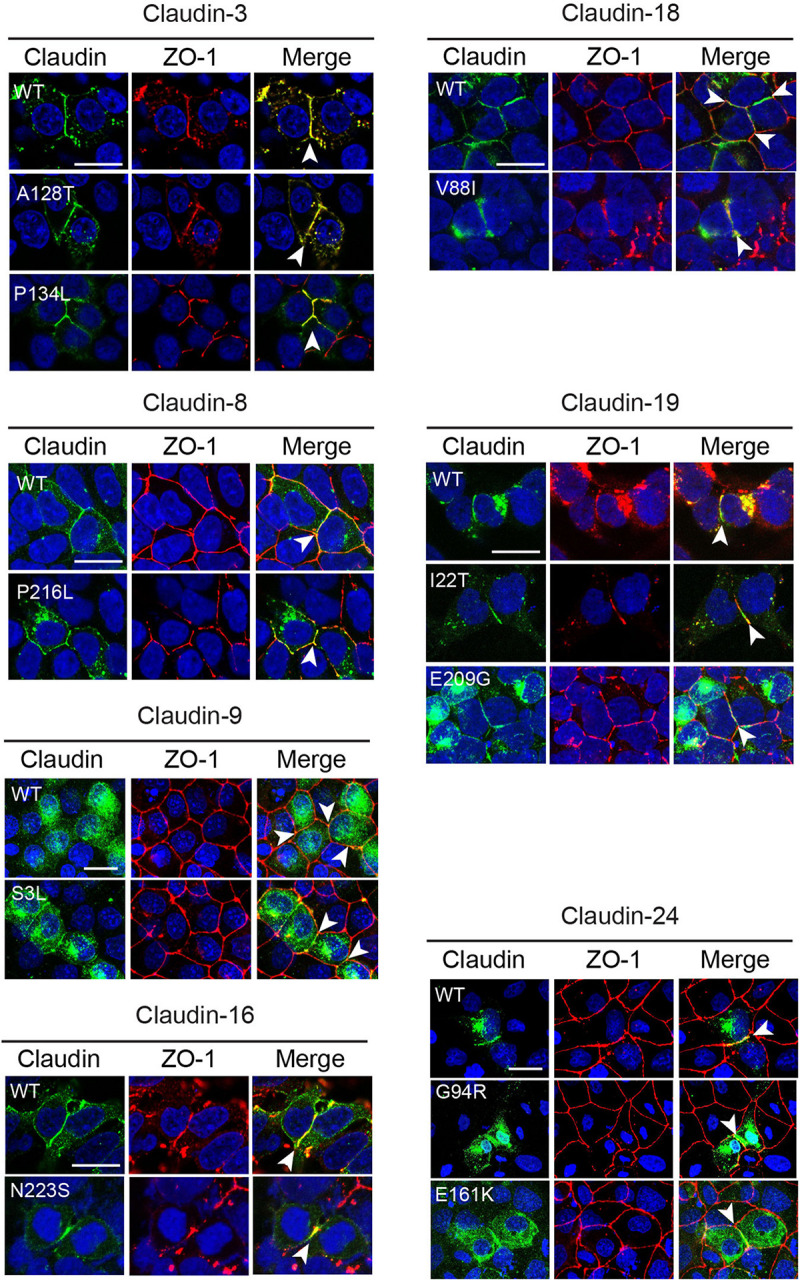
Subcellular localization of CLDN variants. Wild-type (WT) and variant claudins (green) co-localized with the tight junction adaptor protein ZO-1 (red) at the cell membrane (arrowhead) in transiently transfected HEK293 (CLDN3, 4, 8, 16, 18, and 19) or MDCK II cells (Cldn9 and 24). Cldn3, -8, and -16 were detected with anti-claudin antibodies. Cldn9, -18, -19, and -24 were detected with an anti-HA antibody. Blue indicates DAPI stain of the nucleus. The images shown are maximum intensity projections. Scale bar, 20 μm.

Claudins are able to localize to cell–cell contacts and induce the formation of tight junction strands when transfected in tight junction-free HEK293 cells (Milatz et al., 2015; Piontek et al., 2017). To determine if the *CLDN* variants identified in the NTD patients affected the ability of the CLDN protein to localize to the cell membrane, we transiently transfected HEK293 cells with expression vectors containing the wild-type or variant *CLDN* cDNA. The ability of the claudin to co-localize with ZO-1, a classic component of the tight junction cytoplasmic plaque (Stevenson et al., 1986, 1989), was assessed. Wild-type and variant CLDN3, CLDN8, CLDN16, and CLDN19 proteins were able to co-localize with ZO-1 at points of cell–cell contact between neighboring transfected HEK 293 claudin-expressing cells (Figure 3), suggesting that these amino acid substitutions do not impact the ability of variant CLDN to integrate into a tight junction strand. Although the CLDN18 V88I protein was localized to cell–cell contacts, it appeared to be less tightly associated with the membrane than wild-type CLDN18.

Wild-type CLDN9 and 24 protein and their corresponding variants did not localize to the membrane in transiently transfected HEK293 cells (data not shown). To determine if these claudins might require the presence of other claudin family members, we transiently transfected *CLDN9* and *CLDN24* cDNA expression vectors into MDCK II cells that express many claudin family members and which are localized in tight junctions. In MDCK II cells, both wild-type CLDN9 and 24 protein co-localized with ZO-1 at cell contacts. The *CLDN9* S3L and *CLDN24* G94R and E161K variants did not affect the ability of the protein to localize to the tight junction (Figure 3). *CLDN24* E161K was not subjected to further functional analyses because we were unable to confirm by Sanger sequencing due to lack of patient DNA.

These data support that the missense variants were able to co-localize with tight junction proteins similarly to the wild-type protein. Cytoplasmic accumulation of claudin proteins was observed for both wild-type and variant forms of each family member. This is likely to be a consequence of overexpression and not related to the variant.

### Overexpressing *CLDN3*, *CLDN8*, and *CLDN19* Variants Cause Open NTDs in Chick Embryos

To investigate the potential pathogenic role of the rare and novel missense *CLDN* variants identified in NTD patients, we used an overexpression assay in the chick embryo model to examine the effect of these variants on neural tube closure. Previous studies have shown that knockdown or overexpression of NTD-related genes can lead to similar NTD phenotypes (Ting et al., 2003; Yu et al., 2006; Gustavsson et al., 2007; De Castro et al., 2018). The chick embryo is an excellent model to study neural tube closure for several reasons: (1) the tissue and cellular behaviors that regulate neurulation in the chick have been well-studied, (2) the morphogenetic movements regulating neural tube closure in chick and human embryos are predicted to be very similar, (3) neural tube closure occurs over a short period of time (∼28 h) making it easy to collect a large number of embryos (Schoenwolf and Franks, 1984; Smith and Schoenwolf, 1987; Schoenwolf et al., 1988; Schoenwolf and Alvarez, 1989; Schoenwolf, 1991; Smith et al., 1994; Moury and Schoenwolf, 1995; Sausedo et al., 1997; Lawson et al., 2001; Kinoshita et al., 2008; Nishimura et al., 2012; Chen et al., 2017), and (4) the neural tube is easily accessible for manipulation in *ex ovo* culture.

**FIGURE 4:**
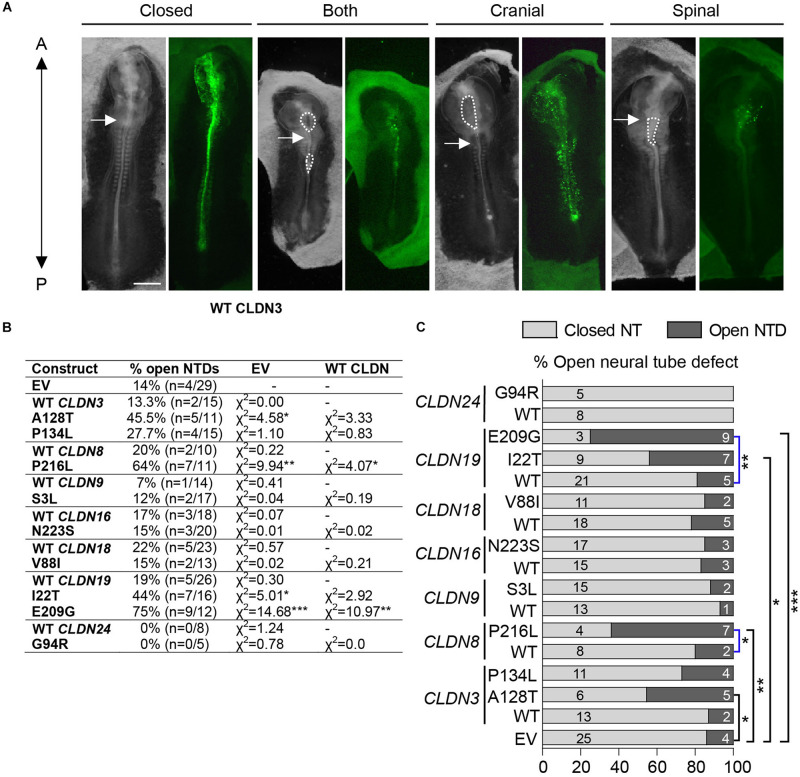
Open NTDs in chick embryos overexpressing CLDN variants. **(A)** Dorsal view of chick embryos electroporated with different Cldn-IRES-GFP constructs showing examples of a closed neural tube, and a complete, a cranial or a spinal NTD; brightfield (left) and GFP expression (right). Closed = *CLDN6* R209Q, both = *CLDN19* I22T, cranial = *CLDN8* P216L, and spinal = *CLDN19* E209G. The anterior end (A) of embryos is at the top of the image and the posterior end (P) at the bottom of the image. Dashed lines outline open neural tubes. Arrows indicate the first somite. Scale bar, 500 μm. **(B)** Overexpression experiments were performed for GFP empty vector (EV), wild-type (WT) Claudins and each Claudin variant construct. Scores for open neural tube defects (NTDs) are shown comparing Claudin variants to wild-type Claudin or empty GFP vector. **(C)** Graphical representation of (C) showing the proportion of open NTDs in embryos injected and electroporated with GFP empty vector (EV) or overexpressing wild-type (WT) or variant Claudin constructs. Light gray bars indicate the number of normal closed neural tubes. Dark gray bars indicate the number of open NTDs. Blue brackets compare wild-type *CLDN*s to *CLDN* variants and black brackets compare empty vector to *CLDN* variants. EV indicates empty vector control, (*p < 0.05, **p < 0.005, ***p < 0.0005, Chi-square test).

Neural plate stage chick embryos (HH4) were injected and electroporated with GFP expression vectors encoding wild-type or variant human *CLDN*s and cultured *ex ovo* until they reached HH11-13, when they could be assessed for NTDs. A range of NTD phenotypes was observed varying from an opening in the future brain (cranial), an opening in the future spinal cord (spinal) or openings in the future brain and spinal cord (both) (Figure 4A). Our background level of NTDs due to experimental manipulation was 14% (*n* = 4/29) as determined by the electroporation of an ‘empty’ expression vector that expressed only GFP. Overexpression of wild-type *CLDN*s did not significantly affect neural tube closure as compared to control injected and electroporated embryos (*p* > 0.05, Figures 4B,C) suggesting that increased expression of wild-type *CLDN*s do not disrupt normal morphogenesis of the neural tube. Although predicted to be deleterious by *in silico* analysis, overexpression of *CLDN3* P134L, *CLDN9* S3L, *CLDN16* N223S, *CLDN18* V88I, and *CLDN24* G94R did not increase the incidence of NTDs as compared to wild-type *CLDN3* (χ^2^ = 0.83, *p* = 0.361), *CLDN9* (χ^2^ = 0.19, *p* = 0.665), *CLDN16* (χ^2^ = 0.02, *p* = 0.888), *CLDN18* (χ^2^ = 0.81, *p* = 0.389) and *CLDN24* (χ^2^ = 0.0, *p* = 0.0) or control embryos (Figures 4B,C). In contrast, overexpression of *CLDN3* A128T caused a significant increase in open NTDs compared to empty vector control embryos (χ^2^ = 4.58, *p* = 0.0323) and three times as many NTDs compared to wild-type *CLDN3* (χ^2^ = 3.33, *p* = 0.0681) (Figures 4B,C). *CLDN8* P216L also caused a significant increase in open NTDs compared to controls (χ^2^ = 9.94, *p* = 0.00162) and wild-type *CLDN8* (χ^2^ = 4.07, *p* = 0.0436) (Figures 4B,C). Overexpression of *CLDN19* I22T caused a significant increase in NTDs compared to empty vector control embryos (χ^2^ = 5.01, *p* = 0.025) and a twofold increase in the incidence of NTDs compared to wild-type CLDN19 (χ^2^ = 2.92, *p* = 0.088) (Figures 4B,C). Finally, a significant increase in open NTDs was observed in embryos electroporated with *CLDN19* E209G compared to both wild-type *CLDN19* (χ^2^ = 10.97, *p* = 0.000924) and empty vector control embryos (χ^2^ = 14.68, *p* = 0.000127) (Figures 4B,C).

**FIGURE 5:**
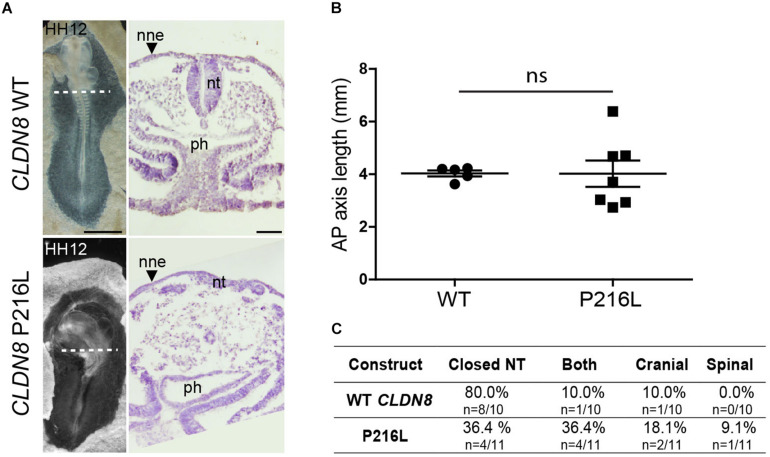
Embryos overexpressing *CLDN8* P216L exhibit open NTDs due defective apical constriction. **(A)** Left, dorsal view of chick embryos electroporated with wild-type (WT) *CLDN8* or *CLDN8* P216L. Right, H&E staining of transverse sections of whole embryos at the level indicated by the dashed line. Scale bars, 1 mm (left) and 50 μm (right). **(B)** Graph shows anterior–posterior (AP) length of embryos electroporated with wild-type (WT) *CLDN8* and *CLDN8* P216L (mean ± s.e.m., ns, non-significant, Mann–Whitney *U*-test). **(C)** Table showing the distribution of complete, cranial and caudal open NTDs observed in embryos overexpressing wild-type *CLDN8* or *CLDN8* P216L. nne, non-neural ectoderm; nt, neural tube; ph, pharynx.

**FIGURE 6:**
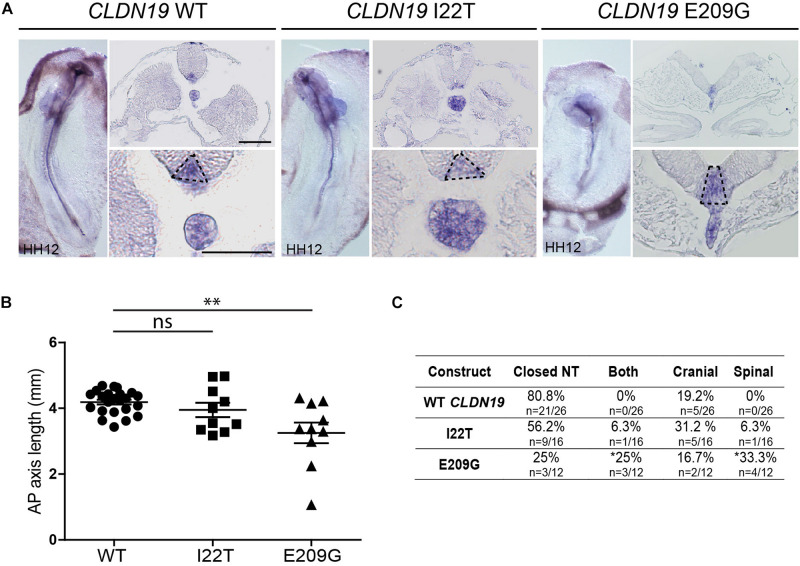
Embryos overexpressing *CLDN19* E209G exhibit open NTDs due to convergent extension defects. **(A)** Left, whole mount *in situ* hybridization for *Shh* of embryos electroporated with wild-type (WT) *CLDN19*, *CLDN19* I22T and *CLDN19* E209G. Right, transverse sections through whole embryos. Dashed line in higher magnification view marks *Shh* expression domain in floor plate. Scale bar, 50 μm. **(B)** Graph shows anterior–posterior (AP) length of embryos electroporated with wild-type *CLDN19*, *CLDN19* I22T and *CLDN19* E209G at the neural tube stage (mean ± s.e.m., *p < 0.05, **p < 0.005, Mann–Whitney *U*-test). **(C)** Table showing the distribution of complete, cranial and caudal open NTDs observed in embryos overexpressing wild-type *CLDN19*, *CLDN19* I22T or *CLDN19* E209G (*p < 0.05, Chi-square test).

To identify the specific events of neural tube morphogenesis affected by overexpressing the *CLDN* variants, we investigated their effects on convergent extension and apical constriction. Convergent extension defects are manifested by a shortened and wider body axis, while a broadened neural tube midline is characteristic of defective apical constriction. Histological analysis of embryos overexpressing *CLDN8* P216L revealed that the midline of the neural tube was flat and the neural folds failed to elevate at the level of open NTDs (Figure 5A). These data suggest that this variant impedes apical constriction of midline cells, which is essential for formation of the median hinge point and consequently elevation of the neural folds. However, they were of normal length compared to wild-type *CLDN8* overexpressing embryos (*p* = 0.8763), indicating that convergent extension was not affected (Figure 5B). Finally, both cranial and spinal open NTDs were observed in these embryos (Figure 5C). In contrast, the NTDs observed in embryos overexpressing *CLDN19* E209G appear to be due to defects in convergent extension. These embryos were significantly shorter than embryos overexpressing wild-type *CLDN19* (*p* = 0.0015), but apical constriction to form the median hinge point appears to have occurred normally as evidenced by the wedge-shaped *Shh* expression domain at the neural tube midline (Greene et al., 1998; Ybot-Gonzalez et al., 2007) (Figures 6A,B). Overexpression of *CLDN19* E209G generated a significant increase in spinal (*p* = 0.0019) and both cranial and spinal NTDs (*p* = 0.0079) compared to wild-type *CLDN19* (Figure 6C).

**FIGURE 7:**
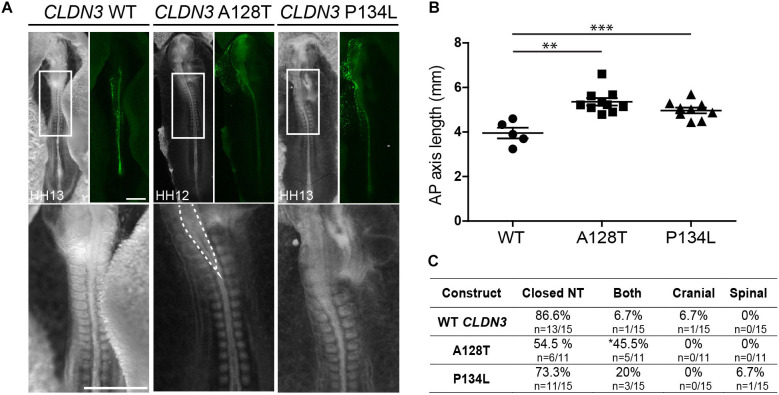
Embryos overexpressing *CLDN3* A128T exhibit open NTDs. **(A)** Dorsal view of chick embryos electroporated with wild-type (WT) or variant Cldn3-IRES-GFP constructs. Brightfield images are shown on the left and GFP expression on the right. High magnification images of the boxed region are shown in the bottom row. Dashed lines outline the open neural tube of an embryo overexpressing *CLDN3* A128T. Scale bar, 500 μm. **(B)** Graph shows anterior–posterior (AP) length of embryos electroporated with wild-type *CLDN3*, *CLDN3* A128T and *CLDN3* P134L at the neural tube stage (mean ± s.e.m., **p < 0.005, ***p < 0.0005, Mann–Whitney *U*-test). **(C)** Table showing the distribution of complete, cranial and caudal open NTDs observed in embryos overexpressing wild-type *CLDN3*, *CLDN3* A128T or *CLDN3* P134L (*p < 0.05, Chi-square test).

In contrast to the severe NTDs observed when convergent extension or apical constriction events are impacted, the NTDs observed in embryos overexpressing *CLDN19* I22T and *CLDN3* A128T were relatively mild, with small openings along the entire length of the neural tube, suggestive of defects in neural fold fusion (Figures 6A, 7A). Although the *CLDN3* A128T embryos were somewhat longer than embryos electroporated with wild-type *CLDN3* (*p* = 0.0007), they were not longer than those overexpressing *CLDN3* P134L (*p* = 0.1023), which did not cause a significant increase in the incidence of NTDs (Figures 4B, 7B). Overexpression of *CLDN3* A128T caused a significant increase in both cranial and spinal NTDs (*p* = 0.0204) compared to wild-type *CLDN3* (Figure 7C). Similarly, *CLDN19* I22T embryos did not exhibit apical constriction nor convergent extension defects. A cross section through the neural tube of these embryos revealed that the neural folds elevated but failed to fuse suggesting that the NTDs are caused by defective fusion of the neural folds (Figure 6A). Together, these data indicate that the novel p.A128T variant in *CLDN3* and the rare variants p.P216L in *CLDN8* and p.I22T and p.E209G in *CLDN19* identified in our NTD cohort are pathogenic.

## Discussion

Previously we showed that depletion of Cldn3, -4, and -8 from tight junctions in the neural (Cldn4 and -8) and non-neural ectoderm (Cldn3 and -4) results in NTDs in chick and mouse embryos (Baumholtz et al., 2017). Morphological analysis of chick embryos revealed that these claudins play critical roles in regulating convergent extension and apical constriction, two morphogenetic events that drive neural tube closure (Baumholtz et al., 2017). However, the possible contribution of rare and novel mutations in *CLDN* genes to human NTDs has not yet been studied. Using a targeted high-throughput sequencing strategy for *CLDN* genes, we identified 17 common, 11 rare and four novel variants in a cohort of 152 NTD patients with the open spinal NTD myelomeningocele. Eight of the rare variants (*CLDN3* p.P134L, *CLDN8* p.P216L, *CLDN9* p.S3L, *CLDN16* p.N223S, *CLDN18* p.V88I, *CLDN19* p.I22T and p.E209G, and *CLDN24* p.E161K) and two of the novel *CLDN* variants (*CLDN3* p.A128T and *CLDN24* p.G94R) were predicted to be pathogenic by at least one prediction program. Overexpression assays in a chick embryo model demonstrated that *CLDN3* A128T, *CLDN8* P216L, and *CLDN19* I22T and E209G generated a significant increase in open NTDs compared to overexpression of the ‘empty’ vector or the wild-type *CLDN*. All of these variants appear to localize normally to tight junctions at cell–cell contacts, which suggests that they may contribute to NTDs by interfering with protein–protein interactions at the tight junction. Our data suggest that variants in CLDN proteins may underlie a subset of human NTDs.

Pathogenic *CLDN* variants could affect neural tube closure by three possible mechanisms. First, they may function as null alleles due to effects on protein stability, function or tight junction localization and cause NTDs due to insufficient levels of the claudin protein at the tight junction. Second, the variant may lead to the production of a dominant negative protein that could compete with wild-type protein for incorporation into tight junction strands, but once there it would the impede the function of wild-type claudins. Finally, the variant may predispose an individual to having an NTD but be insufficient to independently cause an NTD. In these cases, we would predict that a ‘second hit’ such as a variant in another claudin family member or a component of an intersecting pathway would be required to induce NTDs.

None of the *CLDN* variants that we identified in this myelomeningocele cohort were truncating alleles nor predicted to generate a null allele. Moreover, we did not expect a claudin null allele to cause an NTD given that none of the *CLDN* null mice have NTDs (Gow et al., 1999; Furuse et al., 2002; Ben-Yosef et al., 2003; Nitta et al., 2003; Kitajiri et al., 2004; Anderson et al., 2008; Tamura et al., 2008; Nakano et al., 2009; Muto et al., 2010; Ding et al., 2012; Fujita et al., 2012; Hayashi et al., 2012; Kage et al., 2014; Kooij et al., 2014; Li et al., 2014; Matsumoto et al., 2014; Ahmad et al., 2017; Bardet et al., 2017). Although a *Cldn8* null mouse has not yet been reported, *Cldn3* (Kooij et al., 2014; Ahmad et al., 2017; Bardet et al., 2017) and *Cldn19* null mice (Miyamoto et al., 2005) have closed neural tubes. This is perhaps not surprising as knockout or knockdown of a single claudin often results in compensatory upregulation of another family member (Li et al., 2014). Indeed previous data from our lab showed that depleting Cldn4 led to an increase in the level of Cldn8 in the neural ectoderm that is predicted to be compensatory, as the Cldn4-depleted chick embryos developed normally (Baumholtz et al., 2017). In addition, all were able to co-localize with ZO-1 at tight junctions in transfected cells as effectively as the wild-type claudin, suggesting that they do not impede the ability of the claudin protein to localize to tight junctions. However, it is possible that these variant alleles affect the tight junction barrier and future studies are needed to address this question.

Our overexpression functional assay in chick embryos is most effective at identifying variants that have the potential to act as ‘dominant negatives’ or ‘competitive inhibitors’ since we are expressing them in the context of a full complement of wild-type claudins. We noted that overexpression of wild-type *CLDN*s did not cause NTDs suggesting that higher levels of a wild-type claudin protein are not pathogenic. We tested the functional pathogenicity of nine variants that were predicted to be pathogenic by one or more analysis software programs. Four of these variants caused open NTDs when overexpressed in chick embryos, while overexpression of the corresponding wild-type *CLDN* did not. Interestingly, the distinct phenotypes observed when pathogenic variants were overexpressed in chick embryos correlate with the position of the variant in the claudin protein and suggest that claudins function in multiple phases of neural tube closure. *CLDN19* I22T and *CLDN3* A128T, which are in the first and third transmembrane domains, respectively, caused a significant increase in NTDs in chick embryos due to defects in the fusion of the neural folds to form a closed neural tube. Both variants change the amino acid in the transmembrane domain to a threonine, which has the potential to disrupt heteromeric *cis-*interactions between claudins and the morphology of tight junction strands (Rossa et al., 2014). In addition, *CLDN19* p.I22T, but not *CLDN3* p.A128T, introduces a putative phosphorylation site that could induce a further conformational change. These alterations to tight junction morphology could affect tight junction barrier properties.

The mechanism by which *CLDN3* and *CLDN19* could regulate neural fold fusion remains unknown, but may relate to their function in regulating the tight junction barrier. In addition, *Cldn3* expression is limited to the non-neural ectoderm (Simard et al., 2006; Baumholtz et al., 2017), which functions primarily in neural fold fusion. The initial contact between apposing neural folds is mediated by extracellular matrix proteins secreted from the neural and non-neural ectoderm in the chick embryo. It was shown that Cldn3 and -19 are located in tight junctions of ameloblasts in the mouse tooth germ where they play a role in regulating extracellular pH, which is critical for processing and secretion of extracellular matrix proteins (Bardet et al., 2017; Yamaguti et al., 2017). *Cldn3* knockout mice exhibit enamel defects caused by excess accumulation of extracellular matrix proteins in the forming enamel and mutations in *CLDN19* are associated with Amelogenesis Imperfecta, a genetic disorder characterized by tooth enamel defects (Bardet et al., 2017; Yamaguti et al., 2017). The *CLDN3* p.A128T and *CLDN19* p.I22T mutations located in the transmembrane domains of these barrier and pore-forming claudins, respectively, could affect the tight junction barrier leading to a pH imbalance in the extracellular space and lack of secretion of extracellular matrix proteins that are critical for mediating the initial contact between apposed neural folds. The hypothesis will be explored in future studies to understand the mechanisms by which these claudins affect the tight junction barrier.

More dramatic open NTDs due to failures in apical constriction and convergent extension were caused by pathogenic variants in the cytoplasmic C-terminal domain, *CLDN8* P216L and *CLDN19* E209G, respectively. The NTDs in the *CLDN8* P216L and *CLDN19* E209G embryos closely resembled those observed in the embryos that were depleted for Cldn3, 4 and 8. Apical constriction and convergent extension are both early steps in neural tube morphogenesis that depend on cell-shape remodeling and cell–cell interactions. The claudin C-terminal cytoplasmic domain interacts directly with proteins in the tight junction cytoplasmic plaque, which interacts with the actin-cytoskeleton and is a docking site for several regulatory proteins. The *CLDN8* p.P216L variant is likely to alter the conformation of the cytoplasmic C-terminal tail, while the *CLDN19* p.E209G variant results in loss of a negatively charged amino acid immediately adjacent to the cytoplasmic C-terminal PDZ-binding domain. Both of these variants have the potential to disrupt interactions with the cytoplasmic tail, including PDZ-domain adaptor proteins that connect the tight junction to the actin cytoskeleton. In particular, the loss of a negatively charged amino acid is predicted to destabilize interactions with partners that play critical roles in neural tube morphogenesis.

The myelomeningocele patient in our study cohort with the *CLDN8* p.P216L variant was of African ancestry. Given that the MAF is >3% in both African Americans in the EVS database and Africans in the gnomAD database, we would predict that this variant alone is not sufficient to cause NTDs in humans. However, we cannot rule out that it is a risk factor and could predispose the embryo to develop an NTD in the context of a second genetic hit or an environmental insult.

One limitation to our functional validation assay in the chick embryos is our inability to test whether the remaining 5 pathogenic variants generate hypomorphic alleles that may confer significant risk for NTDs in the context of a second mutation or environmental insult. Components of the PCP pathway have a well-established role in regulating convergent extension movements during neural tube closure (Greene et al., 1998; Goto and Keller, 2002; Wallingford and Harland, 2002; Curtin et al., 2003; Wang J. et al., 2006; Wang Y. et al., 2006; Ybot-Gonzalez et al., 2007; Paudyal et al., 2010) and mutations in PCP pathway genes have been recognized as risk factors for human NTDs (Kibar et al., 2009, 2011; Lei et al., 2010; Bosoi et al., 2011; Allache et al., 2012; De Marco et al., 2012, 2013; Robinson et al., 2012). We previously showed that claudins act upstream of PCP signaling and convergent extension during neural tube closure (Baumholtz et al., 2017). Interestingly, four patients with a rare or novel mutation in a *CLDN* gene also carried a PCP gene variant. Only one of these four *CLDN* variants caused NTDs in our overexpression assay (*CLDN3* p. A128T) in the chick embryo. However, it is possible that all four act synergistically with the PCP variants, thereby conferring a high risk for NTDs – a two-hit model for NTDs. This digenic model for NTDs has been observed in mice carrying mutations in PCP genes where homozygous mice have a closed neural tube (e.g., *Fzd3^–/–^* or *Fzd6^–/–^*) (Wang et al., 2002; Guo et al., 2004), while double homozygous mice (e.g., *Fzd3^–/–^;Fzd6^–/–^*) develop NTDs (Wang Y. et al., 2006). Furthermore, heterozygous mice with spinal NTDs (e.g., Vangl2^*Lp/*+^) develop craniorachischisis, a severe NTD that affects the entire embryonic axis, when crossed to heterozygous mice carrying a mutations in a second PCP gene (e.g., *Vangl2^*Lp/*+^; Scrib^*Crc/*+^*, *Vangl2^*Lp/*+^; Celsr1^*Crsh/*+^*, *Vangl2^*Lp/*+^; Ptk7^*chz/*+^*) (Murdoch et al., 2014). It would be interesting to determine if there are genetic interactions between the *CLDN* and *PCP* variants detected in the same patient. In addition, the remaining patients should be screened for mutations in PCP genes.

The mechanisms involved in spinal NTDs are thought to differ from those of cranial NTDs; apical constriction is more important for cranial neural tube closure, while convergent extension plays a more critical role in spinal neural tube closure in chick embryos. Fusion of the neural folds to form a closed neural tube occurs at the cranial and spinal levels. We have shown that claudins are required for apical constriction and convergent extension and thus, mutations in *CLDN* genes may be implicated in both cranial and spinal NTDs. In the future, it would be interesting to sequence genomic DNA from patients with cranial NTDs to compare the pathogenesis of rare and novel *CLDN* variants in the different types of NTDs.

## Conclusion

Our study highlights the conserved function of claudins among species in neural tube closure and supports the hypothesis that rare deleterious variants in *CLDN* genes are significant risk factors for human spinal NTDs. Furthermore, our study demonstrates the importance of validating pathogenicity in a biological system rather than strictly relying on predictive programs: while *CLDN3* p.P134L, *CLDN9* p.S3L, *CLDN18* p.V88I, and *CLDN24* p.G94R were predicted to be pathogenic by all three prediction program, they did not affect the ability of the Cldn protein to localize to tight junctions or cause NTDs in our overexpression assay. In contrast, the *CLDN8* p.P216L variant was predicted to be benign SIFT and MutationTaster, yet overexpression of this variant caused a significant increase in NTDs in our overexpression assay. This highlights the limitation of relying on *in silico* prediction programs to predict pathogenicity of non-synonymous variants which exploit limited information such as amino evolutionary conservation and properties, and do not consider known protein functional domains or tissue-specific protein functions. To our knowledge, this is the first study to identify deleterious variants in *CLDN* genes in patients with NTDs, which further supports the concept that human NTDs are a group of disorders with high genetic heterogeneity, and both *CLDN* and PCP genomic variants contribute to their occurrence. These results enhance our understanding of the pathogenesis of NTDs in humans, highlighting the importance of considering variants in the claudin family as new candidates for NTDs in humans.

## Data Availability Statement

The datasets for this article are not publicly available because of ethical and legal reasons. Requests to access the datasets should be directed to VC at valeriacapra@gaslini.org.

## Ethics Statement

The studies involving human participants were reviewed and approved by IRCCS Istituto Giannina Gaslini (Protocol number: IGG-VACA, 18 September 2011) Research Institute of the McGill University Health Centre (Protocol number: 14-444-PED). Written informed consent to participate in this study was provided by the participants’ legal guardian/next of kin.

## Author Contributions

AB and AR conceptualized the study. AR supervised the implementation and helped with data analysis and drafting the manuscript. AB conducted the cell and chick functional experiments, performed the bioinformatic data analysis, and participated in drafting the manuscript. VC provided critical comments of the manuscript and participated in subject enrollment. PD participated in subject enrollment and extracted genomic DNA from the blood of enrolled patients. All authors contributed to the article and approved the submitted version.

## Conflict of Interest

The authors declare that the research was conducted in the absence of any commercial or financial relationships that could be construed as a potential conflict of interest.

## Footnotes

1 https://varnomen.hgvs.org/

2 http://evs.gs.washington.edu/EVS/

3 https://gnomad.broadinstitute.org/

4 http://genetics.bwh.harvard.edu/pph2/

5 http://sift.bii.a-star.edu.sg/

6 http://www.mutationtaster.org/

7 https://www.ebi.ac.uk/Tools/msa/clustalo/

8 https://www.uniprot.org/

9 http://www.cbs.dtu.dk/services/NetPhos/
