# Supplementary material for: Functional Validation of CLDN Variants Identified in a Neural Tube Defect Cohort Demonstrates Their Contribution to Neural Tube Defects
Source: Front Neurosci. 2020 Jul 14;14:664. doi: 10.3389/fnins.2020.00664 (PMC7372130; doi:10.3389/fnins.2020.00664)
Supplement: Supplementary file 1 [file Table_1.DOCX]

**Supplementary Material**

**
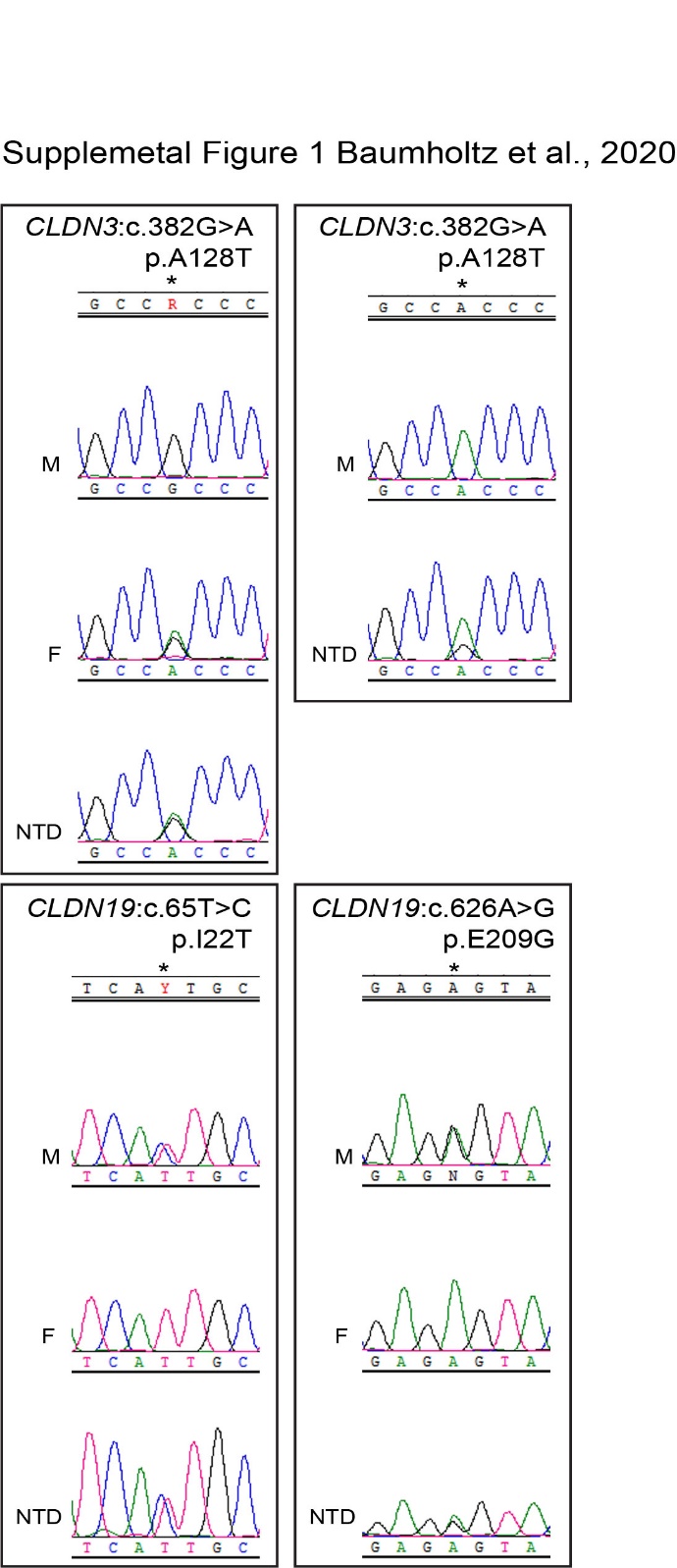
**

Supplementary Figure 1. Chromatograms of rare and novel nonsynonymous *CLDN* mutations in patients and their parents.

Mutations are shown in the sense (5’→3’) direction. The position of altered amino acids are indicated with an asterisk. M, mother’s sequence; F, father’s sequence; NTD; patient sequence.

Supplementary Table 1. Fluidigm primers

| **Fragment** | **Forward primer sequence** | **Reverse primer sequence** | **Fragment size (bp)** |
| --- | --- | --- | --- |
| CLDN1E01 | CCATGGAATCACACAACAGAA | AACTCTCCGCCTTCTGCAC | 371 |
| CLDN1E02 | TGTTTGCAGTTTGCCTTAGA | TTCCATTTTTCTCTTGTTGGTC | 340 |
| CLDN1E03 | ATGGCACTAGCAGGACTTTG | TGGACTTCTAATCTCCCTAATACC | 315 |
| CLDN1E04 | TCCATTTTCGGTTTGTTTCA | CAGAAATCTTAAAGTACTTCCCAAGG | 358 |
| CLDN2E02_001 | GGTGTTCAAGGAGCAAGAGC | GGAGGAGATTGCACTGGATG | 352 |
| CLDN2E02_002 | GCATCACCCAGTGTGACATC | ATGAGCAGGAAAAGCAGAGG | 381 |
| CLDN2E02_003 | TTCTTCCCTGTTCTCCCTGA | CATCCTTGGCAATTCAACCT | 386 |
| CLDN4E01_001 | AGCCTTCCAGGTCCTCAACT | CCTCCAGGCAGTTGGTACAC | 365 |
| CLDN4E01_002 | CATCATCGTGGCTGCTCTG | AGCAGAGAGGAACAGAGTGGA | 402 |
| CLDN5E02_001 | CCCCAGGCTTATCCAACG | GAGGCGTGCTCTACCTGTTT | 361 |
| CLDN5E02_002 | GACAATGTTGGCGAACCAG | GTCCGCAGCGTTGGAGAT | 424 |
| CLDN5E02_003 | GCACGCCAGGATCAGACC | CCCTAACTTCAGCTGCCAGA | 374 |
| CLDN6E01_001 | GCCTCCGCATTAGTTCCATA | CATTACATGGCCCGCTACTC | 372 |
| CLDN6E01_002 | TTCTTGGTAGGGTACTCAGAGG | CACGTGCCCTCTGTGTCAT | 415 |
| CLDN6E01_003 | TCCTCCACACAGGTGGTACA | TGCTTCTGTCCCAAACACAG | 370 |
| CLDN7E01 | CCAGCCGACCACTTCCTC | CGTTTGTTTTACTGTAGGGTCTCC | 417 |
| CLDN7E02 | TCAGTATAGTGAGGCCCCAAA | GGCCCAGGTCTTGGACAC | 393 |
| CLDN7E03 | GGACAGGAACAGGAGAGCAG | CCATCTGGGAGGAGCAAG | 353 |
| CLDN7E04 | AGGCCCTTTCAGGCATCTA | CCCTTTGATCCCTACCAACA | 354 |
| CLDN8E01_001 | CAAAGTTTCTTTGGGGTCCA | GTGGTGCTCATCCCTGTGA | 374 |
| CLDN8E01_002 | AGAGCTTCTCCAAGCTCACG | ACTGTGGATGAATTGCGTGA | 348 |
| CLDN8E01_003 | AGCCAGCAGGGAATCATAGA | CTGGCCAGAAGTAGCAAAGC | 376 |
| CLDN9E01_001 | GGGGCTGAGAAGACCTAACC | GGCGAGGAGGAGGATGAC | 414 |
| CLDN9E01_002 | TGTACCACGTGTGTGGAGGA | ATCAGGCCAAGGTCGAAAG | 409 |
| CLDN9E01_002rd | AAGGCCCGTATCGTGCTC | ATCTGGTCATCAGGCCAAG | 387 |
| CLDN10E01_001 | ACAGGGCATGGGTGTGAG | AGGGAAGGAGGGCTGAGG | 437 |
| CLDN10E02 | TTTTTGGAAAACAAACATCCA | TGAGCACAGCCCTAACAAAT | 361 |
| CLDN10E03 | TTTGGCTGGGATTGTATTCAT | TGCATATTTTGCGTATGTGG | 375 |
| CLDN10E04 | TTGGGATGGTCTAATGGCTA | CAGGTCATTTTTGTCTCTTTTTC | 334 |
| CLDN10E05 | ACTTCTTGGGGCAAGAGGAG | AATTATGGGAGGGCCTTGAT | 352 |
| CLDN11E01 | GTACCTGGGCAGGCACTGT | TATCCCGCTCTCTACCCAGA | 366 |
| CLDN11E02 | AGAAGGAGGAAGGGAGATGG | CTTCACTTGGCTTCCTTTCA | 379 |
| CLDN11E03 | TGGAAGCCACAAGTGTGTGTA | CACACTGTGACGAGCAAACC | 405 |
| CLDN12E04_001 | TCTGACTGACAGTACTCCACAAG | CTGAAGGCAGTGTTGCACAT | 374 |
| CLDN12E04_002 | AGTTTGCCCTACCCCTCAG | TGGGTGGATGGGAGTACAAT | 387 |
| CLDN12E04_003 | TTTGAGCCAGTCTTTTCATTTG | TTGGCTTCATTGATTGGTCA | 360 |
| CLDN14E01_001 | CTGCCTCCATTGACAGTCC | TCCTGGACCACCAACGAC | 382 |
| CLDN14E01_002 | GGAGATGAAGCCCAGGTACA | TCCTACCTGAAAGGGCTCTG | 378 |
| CLDN14E01_003 | ATCGGTAGATCTGGCACTGG | TCGGTGACAGAAATAAGTGCAT | 368 |
| CLDN15E01 | TGCATCCTCACGGAAGTACC | TTCCAGTTCCCTAGGGGTTC | 376 |
| CLDN15E02 | TCTGGGGAGTACAGATGAGG | GATGGGAAAGGCTGACAAC | 361 |
| CLDN15E03 | CAGGATGGAGATCAGTGAGG | CTGCTCTGGGACTTGGTG | 372 |
| CLDN15E04 | TAGGCGTTTCTGCCGTATTT | GGCTGGCGTCTCACTTGT | 352 |
| CLDN15E05 | CGGCCCCTGAGGTTACTA | CCTCACTGATCTCCATCCTG | 366 |
| CLDN16E01 | CCACCCGAAACACACTCAG | GGCCTGGATCATGAAAAGAA | 426 |
| CLDN16E02 | GGCTTCAATTGTCAGTGCTT | TTTTCTGTCCCTTTCCCTTC | 342 |
| CLDN16E03 | AGGGGTACTTATGTTCAAGTTCAT | AGCAGCTTCAGCACAACTCT | 403 |
| CLDN16E04 | TGTAGCATCCTCCCTTTCTTT | TGCCCTTGAACAATTGGA | 359 |
| CLDN16E05 | TTTCCCAAGTTCACTGAGTTCT | AAAGAAAAAGTATAGGAGAATCAAACA | 343 |
| CLDN17E01_001 | GCAAGTTCTCCTGCCTCAC | GTGAGCTGGACAGCCAATA | 358 |
| CLDN17E01_002 | GATGGCTGGGTTGTAGAAATC | TTTGAGAGGCTCTGGGAAG | 333 |
| CLDN17E01_003 | ACAAGGAGCTATAGAACTTGCATT | GTTAGGCCAAGTTCAGTCACA | 375 |
| CLDN18E01 | CACCAGCCTCTCAGAGAAAA | GTTTCCTCTCCACCTCCAAT | 373 |
| CLDN18E02 | TTTGTCTGCTTGTGTCTTGC | TTGGACCTCCACACTCAGAT | 341 |
| CLDN18E03 | CAATATTCTGCAGCCTACTCATC | ATGGCATGGTTGTCTCTGAT | 354 |
| CLDN18E04 | ACCATATTGACAGCCACCAT | GGCTGAAATATTCCCATTCTG | 323 |
| CLDN18E05 | CAAAGACATCTACAATCATGGAAT | AGACTGAGGCTAAGACCATTTG | 375 |
| CLDN19E01 | CTGTTCCCACCTCCCATCT | CTGCCTCTGACCCTCCTTCT | 396 |
| CLDN19E02 | GTGCAGAGGCCTAAAGACAA | CTCTCAAGCTGGGCTCCT | 372 |
| CLDN19E03 | TCCAGTGGACAAAGGTCAGT | GGAGACAGCAACCCCATT | 324 |
| CLDN19E04 | CCTGCCTCTGGTGTCTCTCT | CCTGATGCCACTCTCCCTAC | 380 |
| CLDN19E05 | GACAGACCGAATGATACCATGA | GCCACCTACACAGATGGTGA | 352 |
| CLDN20E02_001 | AGAATTCTGACAGCCATCATTG | TGTCCCCTCCTAAGCGAGTA | 398 |
| CLDN20E02_002 | GCTTTGGGGATCTGCACTT | GCACCCAAAAGTTCCATTTC | 426 |
| CLDN20E02_004 | TCATTTCTGCAATGCTGTTG | AAACATGGAAACAATAATGGAAA | 375 |
| CLDN22E01_001 | GGGACATCCTACCAAATCCA | TGATCCTGGGAGGAATTCTG | 375 |
| CLDN22E01_002 | TCTCATCCCAGAACTCCTGAA | CTTTGCTGGGATGGGTTTTA | 405 |
| CLDN22E01_003 | ACACAGGTTTGCCAGAGTCC | ACCTTGGCATTACTGGTTGG | 379 |
| CLDN23E01_001 | CGACAGCGGAGAAGGAAG | CACGAAGTTGGGCTCGTC | 391 |
| CLDN23E01_002 | GTCCTGGGGCTTCTGCTG | GGCCGTCGCTGTAGTACTTG | 409 |
| CLDN23E01_003 | AGCAGCGTCAGCACCATC | GAAAGGCAGATTTCCATCCA | 394 |
| CLDN24E01_001 | ACATCTCTGGCCTTGTCG | TTTGAGAATTGGAGAGAGTCAGA | 375 |
| CLDN24E01_002 | CAGAATTCCTCCCAGAATGA | TGGCTTTAATCTTTAGAACAGCA | 374 |
| CLDN24E01_003 | TGGCAAATAAGTTGTAATAATGGA | AGCCCTCTTTGTTCATCCTT | 393 |
| CLDN25E01_001 | TTGGACACACCCTCTAAACC | CTCCCAAATCCCCATGAT | 349 |
| CLDN25E01_002 | TGGGTCTGCTCCTGTGTTAC | GGAGACTGGAAGGAGGGTAG | 354 |
| CLDN25E01_003 | GTCTCCTGGGAAGGACTTTG | GGCAGAAGCCAGTCCTAGAT | 349 |
| CLDN25E01_004 | TATTTTCCTGGCTCTTGGTG | AAAAATACATTTGTTAGACACTGTGC | 363 |
| CLDND1E01 | AGGGAAGTGCGTCAGAGGAG | CTGCAGCAGCCACCTCCT | 346 |
| CLDND1E02 | TTATTCCAAATGCACCCTTT | TCAAACCCAGAACACTTAATCC | 341 |
| CLDND1E03_001 | GGCAATCATTACGTCGTTTT | CAGCATTTGTAATTGCTTGTG | 343 |
| CLDND1E03_002 | TGAATTCATCCCAGATGCTT | AGCTTAATTAAAAACATGCTTGC | 343 |
| CLDND1E04 | ATCTTTGTTCCCCCAACTGT | GAGAACCAGTGGCATCCAT | 328 |
| CLDND1E05 | CAGGAAGAAGCTAAAAGGTAAGTATG | GCATCCTTTTGAATAATGTGCT | 339 |
| CLDND1E06 | TTTCATAATAAAATGGTATATCCACAA | TTTTGTTTTGTTTTCTAAATTTTCC | 370 |

Supplementary Table 2. Primer sequences (5’->3’) used for PCR

| **Gene** | **Forward primer** | **Reverse primer** |
| --- | --- | --- |
| *CLDN3* | CGCATATGTCCATGGGCCTGGAGATC | CGTTAGACGTAGTCCTTGCGGTC |
| *CLDN4* | CGCTTGGAATCCTACGGCCC | GCGCTGAGCTCAGTCCAGG |
| *CLDN6* | CCTGTCCACATGTGGCCTGA | GTTGGGCACTGCCACTTCT |
| *CLDN8* | GGTTCCGAGTTCATTACTACAG | GATTAGGCAGTTAAGAACAGTA |
| *CLDN9* | cgctcctgctggacacagagac | gcggcatctggtcatcaggcc |
| *CLDN14* | GCTTCATTAGGGCTCCGGCTG | TGTGCTGGAACCCCTGCCTC |
| *CLDN16* exon 4 | gcctctgaatcacgccagcc | gcttgtttccactggattggga |
| *CLDN18* exon 2 | CGGCTGTCTCTCAGAGGTTTGG | GCGAACCACATTCAGCAGTAGG |
| *CLDN19* exon 1 | CCAGCTGCTCCTCCCACCTG | GCCTACCGTCCAGGGCGAG |
| *CLDN19* exon 4 | GCGGCTCCAGCCTCCAGCTCC | GCGGGTGCTGCGAGGCTGGC |
| *CLDN19* p.E209G | GCGAATTCCATATGGCCAACTCAGGCCTCC | GCGAATTCTCAGACGTACCCTCGGGGCAG |
| *CLDN23* | CGGGAAGGCAGGCTGCAGGG | GCCCAGGCTCTACAAGCGTCTA |
| *CLDN24* | cgcctgtcgcaatggctttaatc | cgttacacttgaggatctgctg |

Supplementary Table 3. Primer sequences used for mutagenesis

| CLDN16N223S-5’ | ggaacgttctactttggttttgcacagtatatttcttggtatcc |
| --- | --- |
| CLDN16N223S-3’ | ggataccaagaaatatactgtgcaaaaccaaagtagaacgttcc |
| CLDN18V88I-5’ | gatgatcgtaggcatcatcctgggtgccattg |
| CLDN18V88I-3’ | caatggcacccaggatgatgcctacgatcatc |
| CLDN19I22T-5’ | gtggctgggtggcatcactgctagcacagccc |
| CLDN19I22T-3’ | gggctgtgctagcagtgatgccacccagccac |

**Supplementary Table 4. *In silico* prediction of pathogenicity for the rare and novel *CLDN* variants**

| **Gene** | **Nucleotide change** | **Amino acid change** | **PolyPhen-2^a^** | **SIFT^b^** | **MutationTaster**  **Model, Score^c^** |
| --- | --- | --- | --- | --- | --- |
| *CLDN3* | c.382G>A | p.A128T | 0.299 | 0.00 | DC, 0.953 |
|  | c.401C>T | p.P134L | 1.0 | 0.01 | DC, 0.999 |
|  | c.620G>C | p.G207A | 0.00 | 0.38 | P, 0.999 |
| *CLDN4* | c.250G>A | p.V84I | 0.001 | 0.51 | PA, 0.999 |
| *CLDN6* | c.626G>A | p.R209Q | 0.001 | 0.75 | P, 0.999 |
| *CLDN8* | c.647C>T | p.P216L | 0.968 | 0.12 | P, 0.999 |
| *CLDN9* | c.8C>T | p.S3L | 0.997 | 0.01 | DC, 0.949 |
| *CLDN16* | c.668A>G | p.N223S | 0.889 | 0.07 | DC, 0.998 |
| *CLDN18* | c.262G>A | p.V88I | 0.998 | 0.04 | DC, 0.999 |
| *CLDN19* | c.65T>C | p.I22T | 0.998 | 0.00 | DC, 0.999 |
|  | c.626A>G | p.E209G | 0.855 | 0.06 | DC, 0.999 |
| *CLDN23* | c.268G>A | p.A90T | 0.001 | 0.34 | P, 0.999 |
| *CLDN24* | c.280G>C | p.G94R | 1.000 | 0.00 | DC, 0.999 |
|  | c.481G>A | p.E161K | 0.999 | 0.03 | DC, 0.999 |
|  | c.529C>A | p.L177M | 0.095 | 0.08 | P, 0.999 |

The score of the above softwares range between 0 to 1. **^a^**The amino acid substitution is predicted as highly damaging/deleterious when the score is close to 1. ^b^The amino acid substitution is predicted as damaging/deleterious when the score is 0.0 to 0.05. ^c^The amino acid substitution is predicted to fall into four possible types: DC, disease causing or probably deleterious; DCA, disease causing automatic or known to be deleterious; P, polymorphism or probably harmless; PA, polymorphism automatic or known to be harmless. A value close to 1 indicates a highly accurate prediction
